# A Counterintuitive Neutrophil-Mediated Pattern in COVID-19 Patients Revealed through Transcriptomics Analysis

**DOI:** 10.3390/v15010104

**Published:** 2022-12-30

**Authors:** Melih Özbek, Halil Ibrahim Toy, Işil Takan, Seyedehsadaf Asfa, Reza Arshinchi Bonab, Gökhan Karakülah, Panagiota I. Kontou, Styliani A. Geronikolou, Athanasia Pavlopoulou

**Affiliations:** 1 Izmir Biomedicine and Genome Center, Balcova, Izmir 35340, Turkey; 2 Izmir International Biomedicine and Genome Institute, Dokuz Eylül University, Balcova, Izmir 35220, Turkey; 3 Department of Mathematics, University of Thessaly, 35131 Lamia, Greece; 4 Clinical, Translational and Experimental Surgery Research Centre, Biomedical Research Foundation Academy of Athens, 11527 Athens, Greece; 5 University Research Institute of Maternal and Child Health and Precision Medicine, UNESCO on Adolescent Health Care, National and Kapodistrian University of Athens, Aghia Sophia Children’s Hospital, 11527 Athens, Greece

**Keywords:** COVID-19 epidemiology, asymptomatic patients, transcriptomics, virus entry spike absence, machine learning, natural language processing, gene signature, cytokine blunt

## Abstract

The COVID-19 pandemic has persisted for almost three years. However, the mechanisms linked to the SARS-CoV-2 effect on tissues and disease severity have not been fully elucidated. Since the onset of the pandemic, a plethora of high-throughput data related to the host transcriptional response to SARS-CoV-2 infections has been generated. To this end, the aim of this study was to assess the effect of SARS-CoV-2 infections on circulating and organ tissue immune responses. We profited from the publicly accessible gene expression data of the blood and soft tissues by employing an integrated computational methodology, including bioinformatics, machine learning, and natural language processing in the relevant transcriptomics data. COVID-19 pathophysiology and severity have mainly been associated with macrophage-elicited responses and a characteristic “cytokine storm”. Our counterintuitive findings suggested that the COVID-19 pathogenesis could also be mediated through neutrophil abundance and an exacerbated suppression of the immune system, leading eventually to uncontrolled viral dissemination and host cytotoxicity. The findings of this study elucidated new physiological functions of neutrophils, as well as tentative pathways to be explored in asymptomatic-, ethnicity- and locality-, or staging-associated studies.

## 1. Introduction

The severe acute respiratory syndrome coronavirus 2 (SARS-CoV-2) is the etiological factor of the alarming inflammatory disease known as COVID-19. SARS-CoV-2 has a 75–80% genomic similarity to SARS-CoV and a 50% similarity to MERS-CoV [1,2]. Its prevalence reached pandemic heights very quickly, whilst, in global terms, 591,683,619 cases (including 6,443,306 deaths) were confirmed by the World Health Organization as of 19 August 2022. The pathology, as well as the severity of the symptoms, vary depending on the coronavirus variant [3].

The induced pathophysiology of the alpha variant (B.1.17) is characterized by hypercytokinemia, organ dysfunction, and lymphopenia, leading to acute respiratory distress syndrome (ARDS) [4], lung injury [5], an increased neutrophil-to-lymphocyte ratio [6,7], coagulopathy, even multiorgan failure [8,9], and sepsis [10]. The pathogenesis of the delta variant has been attributed to the excessive host immune response, which is mediated by increased levels of proinflammatory cytokines, mainly interleukins IL-1α, IL-1β, IL-6, IL-8, and IL-10 as well as interferon-γ (IFN-γ) and tumor necrosis factor-α (TNF-α), known as a “cytokine storm” [11,12,13]. Of these cytokines, IL-6 was found to be significantly positively correlated with a higher mortality. There is accumulating evidence that IL-6 is a principal inducer of immune dysregulation and a perpetrator of the cytokine storm [14,15,16,17].

The omicron variants have been linked to a milder symptomatology and a lower fatality [18,19]. In addition, the massive vaccination of the population contributed to a higher communal immunity with effectiveness or unknown mismatch [19,20].

However, the scientific community has not completed the decipherment of the complex mechanisms linking infection and disease severity; the most impressive characteristic of this pandemic is the heterogeneity in clinical imaging and pathophysiology. Thus, the patterns underlying the pathology, heterogeneity, and severity of COVID-19 remain underexplored, leading mainly to therapies for COVID-19 infections targeting the cytokine signaling processes and hyperinflammation [21,22].

Consequently, as the knowledge gaps are vast in this newly emerged epidemic, novel technologies and approaches have been exploited. Since the COVID-19 pandemic onset, high-throughput sequencing technologies, such as RNA-sequencing (RNA-Seq), have facilitated the generation of a great amount of gene expression data related to the SARS-CoV-2-induced host transcriptome response. In particular, Liao et al. [23], in a RNA-Seq analysis of the alveolar fluid in nine patients, reported that high macrophage-driven responses and a “cytokine storm” were potentially preventing adequate T-cell responses to SARS-CoV-2 in patients with severe diseases. Ong and colleagues, accordingly, using the same method in the blood segments of three patients, associated the intrapulmonary immune response with systemic changes as they observed high IL-1 pathway cytokines followed by a T-cell decrease [24].

The virulence distribution as well as the tissue/organ injuries are under-/misexplored so far, leading to contradictory immune response patterns. Physicians and researchers acknowledge that more accuracy and precision is needed. Personalized treatment has arisen as a necessity but is inconclusive regarding the whole puzzle of tissue immune responsiveness—infectivity signatures—as disease staging and patient clustering remain unelucidated [25,26].

The human host transcriptome profiling of intra- and extrapulmonary tissues based on the blood immune response to SARS-CoV-2 was investigated to identify a soft tissue immune response pattern and diagnostic gene expression signatures.

To this end, we employed an integrative in silico approach to process, analyze, and interpret publicly accessible gene expression data relevant to the host transcriptional response to SARS-CoV-2 infections, including bioinformatics, machine learning, and natural language processing.

## 2. Materials and Methods

### 2.1. Acquisition of High-Throughput Transcriptome Data

The publicly accessible repository NCBI GEO (Gene Expression Omnibus) DataSets (https://www.ncbi.nlm.nih.gov/gds/; accessed on 10 March 2022) [27,28] was searched for transcriptomics datasets related to human responses to SARS-CoV-2. The criteria for choosing datasets were: (i) gene expression data from SARS-CoV-2-infected and healthy tissue samples and (ii) datasets including ≥ 5000 genes. In this way, two eligible RNA-Seq datasets were obtained. The tissue samples in the datasets were divided into two groups: the SARS-CoV-2-infected tissues, or “COVID-19”, and non-infected tissues, or “control”.

The GEO series of the two data sets, including data extracted from patients infected with alpha variant (B.1.17) of SARS-CoV-2, were as follows:

(i) GSE150316 [25] contained genome-wide transcriptomics data of COVID-19 patients collected at two institutions in the USA, the Massachusetts General Hospital (20 patients) and Columbia University Irving Medical Center (4 patients). The transcriptomes of lung, heart, liver, kidney, and bowel tissue samples were investigated (Table S1). The Illumina MiSeq (Homo sapiens) GPL15520 and Illumina NextSeq 500 (Homo sapiens) GPL18573 platforms were used. 

(ii) GSE167000 [26] included global gene expression data from whole blood samples derived from 65 SARS-CoV-2-positive and 30 SARS-CoV-2–negative individuals collected at the University of Colorado Anschutz Medical Campus (Table S1). The Illumina NovaSeq 6000 (Homo sapiens) GPL24676 platform was employed.

### 2.2. RNA-Seq Data Processing

The raw RNA-Seq reads contained in the FASTQ files were retrieved from their respective Sequence Read Archive (SRA) files by using the SRA Tool Kit version 2.9.6 [29] with the fastq-dump –gzip –skip-technical –readids –dumpbase –clip –split-3 command. Subsequently, the RNA-Seq reads were mapped to the human reference genome GRCh38.p13 with annotation derived from Ensembl version 104 by employing the spliced aligner HISAT2 v.2.1.0 [30] with the hisat2 -p -dta -x {input.index} -U {input.fq} -S {out.sam} parameters. The generated SAM files were converted to binary BAM files with the usage of SAMtools v.1.14 [31] with the samtools sort -@ 10 -o {output.bam} {input.sam} commands. The assembler StringTie v.1.3.5 [32], using the stringtie -e -B -p -G {input.gtf} -A {output.tab} -o {output.gtf} -l {input.label}{input.bam} parameters, was employed for transcriptome normalization, assembly, and quantification. The reconstructed transcripts and transcript abundance estimates were reported in the output GTF file. The gene symbols and gene names were assigned according to the official HUGO Gene Nomenclature Committee (HGNC) [33].

### 2.3. Differential Gene Expression Analysis

For blood and lung tissue samples, differential gene expression analysis of the “COVID-19” and “control” RNA-Seq groups was performed using the edgeR package (version 3.32.0) [34] of the R programming environment v.3.6.1 (https://www.r-project.org; accessed on 28 March 2022). First, the trimmed mean of M-value (TMM) normalization implemented in edgeR was applied to the count data. The negative binomial distribution was used to model the RNA-Seq reads per gene per sample in edgeR. The dispersion in the datasets was estimated with the estimateDisp function. The glmFit and glmLRT functions of the edgeR package v3.32.0 were used to fit the data and compare the two RNA-Seq groups, respectively. The Benjamini–Hochberg (BH) method for controlling the false discovery rate (FDR) was used for *p*-value adjustment [35]. Those DEGs, with an absolute log_2_-fold change (FC) greater than 1 (|log_2_FC > 1|) and a corrected *p*-value ≤ 0.05, were considered statistically significant.

Given the small sample numbers for the heart, liver, kidney, and bowel tissues, a different protocol for differential gene expression analysis was followed. In particular, in order to identify more biologically relevant sets of genes, the rank product (*RP*) method was used based on calculating rank products from experiments in a fast and simple way. This method is based on biologically significant FCs, providing, at the same time, an estimate of the statistical significance. The *RP* method is essentially a nonparametric method for detecting DEGs in microarray experiments [36,37]. The genes are ranked according to their FC, and then analysis is performed separately for upregulated and underregulated genes. For instance, concerning the upregulated gene *g* with *i* ¼ 1, 2, *..*., *k* replicates, the rank product is given by the geometric mean:

$$RP\left(g\right)={\left(\overset{k}{\underset{i=1}{\prod }}r_{g,i}\right)}^{1/k}$$

The *RP* method is available as an R package (RankProd). The use of exact calculation and permutation methods have been proposed to determine the statistical significance when the sample size is small. *RP* is more robust and accurate for sorting genes based on differential expression compared to t-statistics, especially for studies with sample size n < 10 [38]. In this study, FC was calculated, and then the RankProd analysis package in R [36] was used to calculate the ranks and the *p*-values of the genes of each tissue. The FDR value of < 5% was applied as a cutoff to increase the power of our study.

Venn diagrams of the DEGs per tissue were constructed using the online tool Draw Venn Diagram (https://bioinformatics.psb.ugent.be/webtools/Venn/; accessed on 3 April 2022).

To visualize the DEGs, heatmap plots of the DEGs were created using the “pheatmap” package of R (https://CRAN.R-project.org/package=pheatmap; accessed on 5 April 2022) v. 1.0.12.

### 2.4. Principal Component Analysis

Principal component analysis (PCA) of the blood and lung transcriptomics data was performed. To this end, blood and lung transcriptomics FPKM data were filtered so the expression value of the genes was greater than “1” in at least half of the samples in each group. Next, the filtered FPKM data were log2-transformed and “1” was added (FPKM + 1) because expression values with “0” cannot be log-transformed. The R “stats” (version 4.1.1) function *prcomp* was used to generate PCA objects of the filtered log2-transformed FPKM values. To visualize the PCA plots, the *fviz_pca_ind* function of the “factoextra” package (https://CRAN.R-project.org/package=factoextra; accessed on 27 March 2022) was used.

### 2.5. Ensemble Machine Learning

To detect the most important DEGs across tissues, a voting classifier, an ensemble model of machine learning, was applied [39]. A voting classifier typically uses multiple models of various types and combines their predictions into a final result with the usage of simple statistics. There are two types of voting, namely hard voting and soft voting. In hard voting, each base model casts one vote for its predicted outcome, and the ensemble model makes a decision on the basis of the majority of the classifiers’ predictions, i.e., if the result of the majority of the models’ votes is class 0, accordingly, the ensemble’s prediction will be class 0 as well. In soft voting, each model outputs a probability for its prediction rather than a single vote, and also the ensemble model takes the classifiers’ average probability per class and makes a prediction based on that average. All voting methods were implemented using the *scikit-learn* library in Python 3.9.7.

First, three classifiers, namely random forest, k-nearest neighbor (KNN), and naïve Bayes, were selected. Random forest is a bagging ensemble algorithm which makes use of multiple different algorithms and generates a final result based on them [40]. KNN [41] classifies a new data point by searching the entire training set for the *k* most similar instances that are closest to the test data point. In KNNs, *k* denotes the number of nearest neighbors that are to be included in the voting processes. Naïve Bayes [41] is a simple yet powerful classification algorithm. It is a conditional probability model, where it is assumed that a value of a particular feature is independent of the value of any other feature, and it is considered to be particularly fast as compared to other more cutting-edge algorithms.

After selecting the classifiers, our dataset, containing 1043 observations, was divided into 75% training data and 25% testing data for all of the models used for classification, and also the random state was set to 42. In addition, the performance metrics accuracy rate and cross-validation were used to assess the proposed model. Accuracy is the measure of how reliable the predictions of the model are, and cross-validation is one of the techniques used to test the effectiveness of machine learning models. After fitting the model and calculating the accuracy, random forest, KNN, and naïve Bayes showed classification accuracies of 98.46%, 92.72%, and 95.01%, respectively. Also, the performance of the hard and soft voting mechanisms was evaluated. The soft voting (probability-based voting) mechanism showed better performance (96.55%) as compared to hard voting. Furthermore, for 10-fold cross-validation, the proposed algorithm achieved accuracies of 95.90% for naïve Bayes, 94.11% for k-nearest neighbor, and 98.08% for random forest.

The genes annotated as pseudogenes in HGNC [33] were not included in the subsequent steps of the analysis.

### 2.6. Functional Enrichment Analysis

To explore biological functions associated with the common DEGs, gene set enrichment analysis was conducted so as to identify relevant gene ontology (GO) terms that were overrepresented in this gene set. To this end, the common DEGs were given as input in WebGestalt (WEB-based GEne SeT AnaLysis Toolkit) [42,43] to identify statistically significant enriched nonredundant GO biological process terms; the threshold for the Benjamini and Hochberg (BH)-adjusted *p*-value [35] was set at 0.05.

### 2.7. Natural Language Processing

The keywords “covid-19” or “sars-cov-2” were used to search the biomedical literature database, MEDLINE/PubMed (https://pubmed.ncbi.nlm.nih.gov/; accessed on 22 February 2022) for scientific publications. Collectively, 149,055 abstracts were obtained, and after the exclusion of publications written in non-English, those with insufficient text for processing, and duplications, a total of 14,862 articles were retained. The abstract of each article was extracted and stored as a JSON file. The spaCy model “en_core_sci_lg” in the scispaCy Python package [44] was employed to retrieve the biomedical entities from the dataset. Gene names were isolated from the processed text and were retained as the final set of documents, containing 4012 articles, so as to define their significance and semantic relationships.

TF-IDF (“Term Frequency-Inverse Document Frequency”), an information extraction subtask, was utilized to signify the importance of the extracted genes in the document. TF-IDF was implemented in Python 3.9.7 using the open-source library *scikit-learn*. The top 50 genes with the highest tf-idf scores were selected for further analysis. Document frequency, the number of occurrences of a term in a document set, was calculated for the first 50 genes.

The gensim *word2vec* module implemented in Python 3.9.7 (Python Software Foundation, Wilmington, DE, USA) (https://www.python.org/; accessed on 26 January 2022) was used to train word vectors of the text, including gene names. Gene-to-gene distances were extracted to calculate the similarity distances between each gene pair using the *word2vec.most_similar* function. The semantic relations of the top 50 genes were detected based on the similarity distances between each gene pair.

## 3. Results

**Figure 1:**
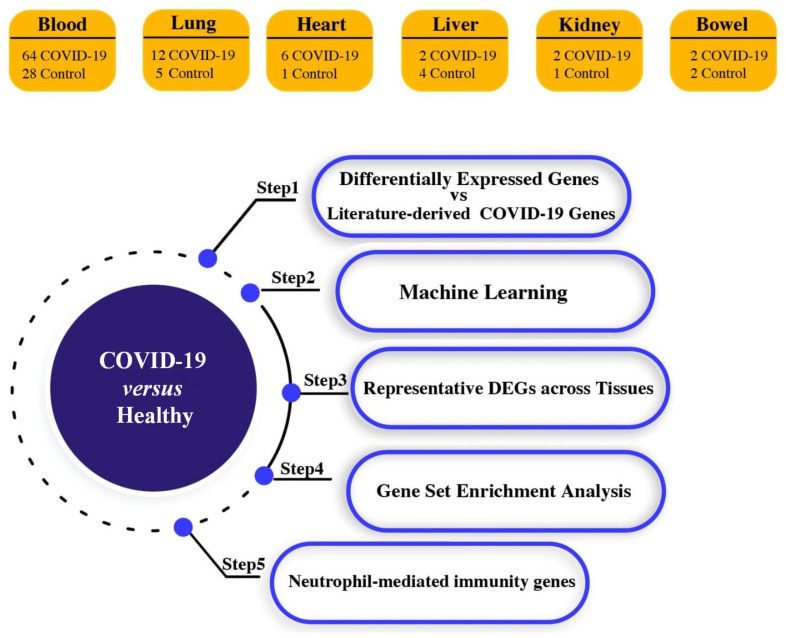
Graphic illustration of the overall methodology of this study.

The workflow of the present study is illustrated in Figure 1.

### 3.1. Identification of Differential Expression Patterns in SARS-CoV-2-İnfected vs. Healthy Tissues

In order to explore the transcriptional dynamics of the human host with respect to SARS-CoV-2 infections, we profited from publicly available RNA-Seq datasets, which were exploited to identify the DEGs of SARS-CoV-2-positive and –negative subjects. To this end, the relevant gene expression data from six different tissues of SARS-CoV-2-positive (COVID-19) and SARS-CoV-2-negative (control) individuals were processed and analyzed by employing an integrated computational methodology.

**Figure 2:**
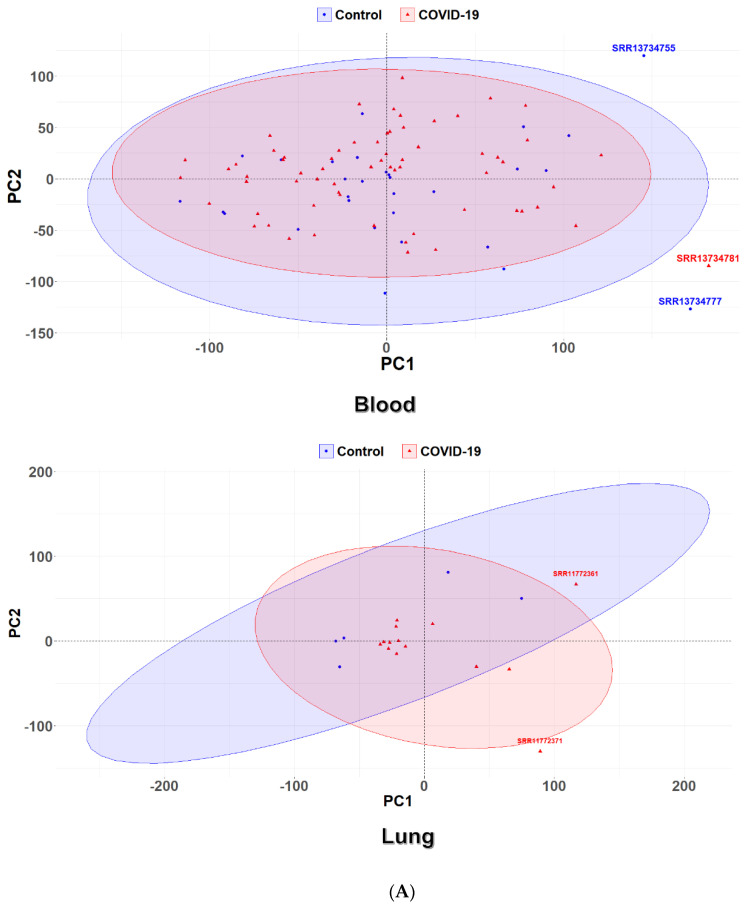

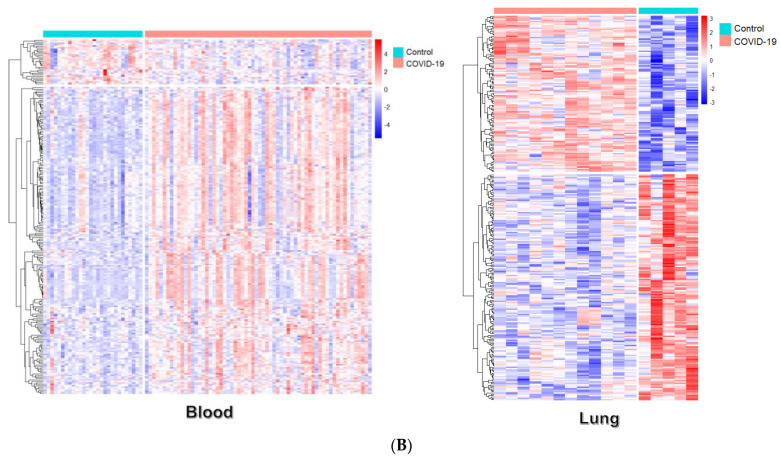
Genes differentially expressed between SARS-CoV-2-infected and control samples. (**A**) PCA of COVID-19 and control samples from blood and lung tissues. Red and blue ellipses represent COVID-19 and control samples, respectively. (**B**) Heatmap of the DEGs in blood and lung tissues. COVID-19 and control samples are shown in salmon and turquoise colors, respectively. Each row corresponds to a DEG. The upregulated and downregulated genes are indicated by red and blue colors, respectively. A dendrogram depicting hierarchical gene clustering (based on Z-scoring) is shown on the left.

The distribution of the COVID-19 and control samples in the blood and lung tissues according to a principal component analysis is shown in Figure 2A. In the blood PCA plot, three outlier samples were detected outside the circle (SRR11734755, SRR11734777, and SRR11734781) which were not included in the subsequent steps of the analysis; similarly, two outliers (SRR11772361 and SRR11772371) were detected in the lung PCA plot. After the removal of the outliers, a total of 64 COVID-19 and 28 control samples were considered for further analysis in the blood tissues, and, also, 12 COVID-19 and 5 control samples were considered in the lung tissue (Table S1). The tissues of the heart, liver, kidney, and bowel had a small number of samples (Table S1), and, thus, a methodology different from the one used for the detection of DEGs in the blood and lung samples was applied.

**Figure 3:**
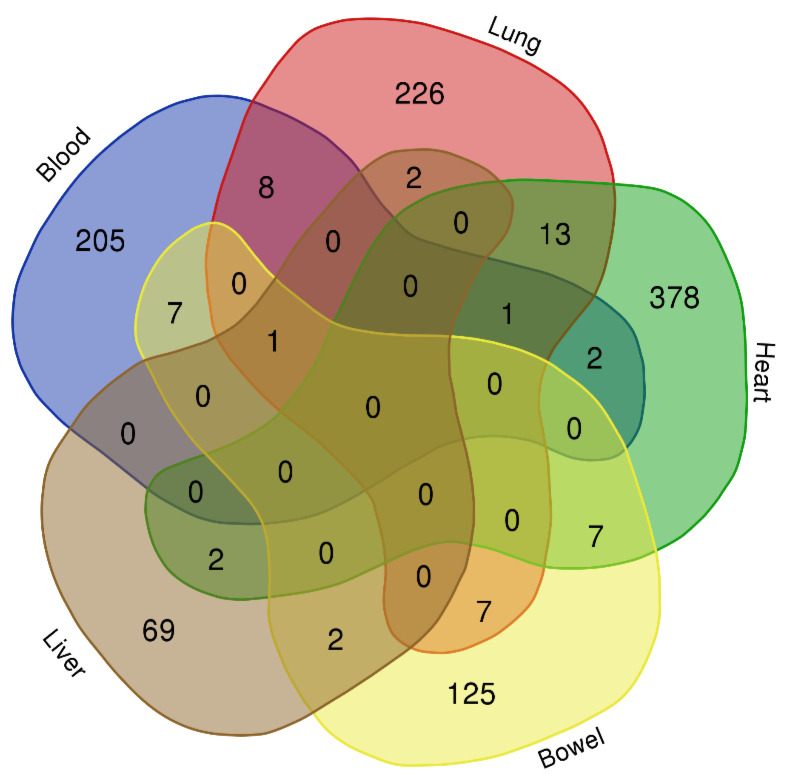
Venn diagram of the overlapping DEGs among tissues.

The number of statistically significant differentially expressed genes (DEGs) found in the “COVID-19” and “control” groups per tissue was 223 (blood), 257 (lung), 403 (heart), 75 (liver), and 148 (bowel). No statistically significant DEGs were detected in the kidney (Figure 2B and Table S2). The total number of unique DEGs across the tissues was 1003 (Table S2). Notably, no significant overlap was observed among the DEGs of the five tissues, and also there were no common genes across all the tissues (Figure 3 and Table 1).

Ensemble learning was applied to select the most important genes that were consistently differentially expressed (i.e., up-/downregulated) across the five tissues. A total of 225 DEGs were detected in this way (Table S3). More impressively, among those genes, contrary to the literature that has been published so far (based on our natural-language-processing-aided thorough literature mining), the expression of the well-studied genes associated with the COVID-19–mediated hyperinflammatory response, such as IL-6, TNF-α, and IFN-γ, were found to be unchanged.

### 3.2. Pathway Enrichment Analysis of Common Selected DEGs

A gene set enrichment analysis was performed to identify overrepresented biological processes in the “prominent” 225 DEGs. A single process was found to be statistically significant which was related primarily to “neutrophil mediated immunity” (GO:0002446) and included 16 genes (Table 2). Neutrophils constitute the most abundant type of white blood cells, and they are essential effectors on the frontline of the host defense against attacking pathogens [45]. Neutrophils contribute to the regulation of inflammation [46]. Recently, apart from their antipathogenic and proinflammatory roles, neutrophils have been suggested to have the ability to suppress the immune response via different mechanisms [47,48].

## 4. Discussion

Clinical complexity and heterogeneity were accounted amid the main problems physicians and researchers had to resolve during the COVID-19 pandemic. Studies on the tissue-specific distribution of genes and their corresponding products and expression are limited. Thus, this investigation focused on the blood and soft tissue sample transcriptomes of COVID-19 patients. An in silico approach was employed with the data retrieved from NCBI’s GEO from two previously published investigations by Galbraith et al. and Desai et al. [25,26].

Our findings revealed that, in the populations investigated, the SARS-CoV-2 infection’s high levels of the characteristic “cytokine storm” and the known attachment contributors to virus entry and tropism (angiotensin-converting enzyme 2 (ACE2), angiotensin II receptor type 2 (AGTR2), alanyl aminopeptidase (ANP), ASGR1, Band3, dipeptidyl peptidase IV (CD26), basigin (CD147), CLEC4G, KREMEN1, low-density lipoprotein receptor class A domain-containing protein 3 (LDLRAD3), neuropilin 1 (NRP1), and transmembrane protein 30A (TMEM30A) [49]) were not expressed in any of the tissues examined herein. Given that the source studies reported no demographic or clinical details of the population, it was imperative for us to examine every option that might explain the cytokine, ACE2, and other SARS-CoV-2 entry spike nonexpression in all the tissues reported, therein.

It was established that the wide heterogeneity and complexity of COVID-19 lies mainly in two factors: the “levels of cytokines” and “hypoxia” [50,51,52]. It was evidenced that patients living permanently in mountains manifested lower levels of IL-6 and TNF-α as well as a lower morbidity and mortality [51,52,53]. The anti-inflammatory and cardioprotective feature of higher-altitude locations has been attributed to the lower expression of angiotensin-converting enzyme 2 (ACE2) in populations residing at such altitudes [54] due to natural hypoxia and higher UVA and UVB exposure, which act as natural sanitizers by shortening any virus’s (such as SARS-CoV-2) half-life [55]. More interestingly, the prevailing data did not provide any results of ACE2 expression in any of the tissues involved. On the other hand, it was established that IL-6 was a key player in homeostasis maintenance [56] as well as a proinflammatory biomarker and strong COVID-19 predictor [17,57]. Although the hypothesis that “cytokines increase is involved in high altitudes induced hypoxia” has been propounded [58], our results contradicted this theory, at least in COVID-19 infection coexistence.

The absence of cytokines in the source studies was diverse in terms of seroepidemiology; viz, the sero-low patients and sero-high patients showed diverse intrapatient and interpatient virulence as well as diverse staging as reported by Galbraith et al. [26]. The same study reported stage 1 patients among the population considered. This stage involves asymptomatic carriers (according to the Centers for Disease Control and Prevention (CDC), they account for 10–60% of the infected population (https://www.cdc.gov/coronavirus/2019-ncov/hcp/planning-scenarios.html; accessed on 5 May 2022)), children, and young adults [59]. This subpopulation is not susceptible to antiviral treatment, at least as long as they do not pass into the symptomatic stage. Asymptomatic clinical images compared to other respiratory diseases were discussed in the Mick et al. study in 2020 [60]. The results were associated with a high viral load in the upper airway [61] and/or possible monoclonal antibody medication administration in the early stages [62]. Immunosuppressant treatment, due to potential relevant comorbidities, such as sepsis, cancer, transplantation, corticosteroids such as anti-inflammatories, renal failure, and chronic hemodialysis, might be partly responsible for the proinflammatory blunt as well [62].

Sarma and colleagues (2021) challenged the cytokine storm model as well, particularly in the lower airways of patients, and propounded dexamethasone as a treatment option [63].

Moreover, a recent study linked the photochemistry mechanism with the ethnicity association to COVID-19 epidemiology (staging, virulence, and morbidity): “skin photo-protection and reduced damaging pro-oxidative species from eumelanin photochemistry may be linked to the increased severity of COVID-19 in dark skinned BAME (Black, Asian, Minority ethnic), whilst in the fair- skinned patients, reactive oxygen species generated by PM photolysis and rearrangement (in skin types I-III), unlike those of dark-skin type IV-V and total absence in black skinned patients VI” [64]. In addition, according to the Centers for Disease Control and Prevention (American CDC), the COVID-19 morbidity rate is 2.8 times higher in the Alaskan and Indian subpopulations; their fatality rate is half (1.4) that of the Caucasians in the United States. This might partly explain the results of Galbrraith et al. and Desai et al., as well as our results.

In a study by Remy and coworkers (2020) on critically ill COVID-19 patients [65], a hypothesis diametrically opposed to the prevailing one was proposed, stating that the severity of COVID-19 is rather due to the collapse of the host’s protective immune system and a profound COVID-19-induced suppression of well-known cytokine-storm-related genes as well as the depletion of the effector cells of the immune system, such as CD3^+^/CD4^+^/CD8^+^ T and NK cells. In the same study, they suggested that the severe immunosuppression accounted for the uncontrolled viral replication and spread, leading to organ injury and host cytotoxicity. Several studies also support that COVID-19 patients are more prone to secondary nosocomial-acquired infections [66,67,68,69]. This is mainly due to the fact that the immune system is compromised and cannot prime a sufficient immune response.

The results of our study, in part, corroborated this hypothesis. Therefore, immunomodulatory therapeutic strategies should be developed to boost the weakened host immune response so as to eradicate SARS-CoV-2 and to eliminate infection. The administration of anti-inflammatory effectors, such as IL-7, was shown to reverse lymphopenia and increase CD4^+^/CD8^+^ T-cell proliferation in septic shock [70]. A further understanding of the inflammatory response in diverse populations would add to the implementation of better and more specific/personalized therapeutic and preventive approaches. Future studies evaluating larger samples, more cytokines, and their clinical impacts are of great need.

Neutrophil involvement was dominant in all the tissues studied herein, indicating either that the population sampling had taken place in the primary stages or that a new signaling pathway, meriting future investigation, is revealing.

Neutrophils are the frontline effectors of the innate immune system’s arsenal against pathogen invaders such as SARS-CoV-2 [71]. On account of their having to effectively function even in hypoxic circumstances, they have developed the sense of perceiving oxygen tensions (through prolyl and asparaginyl hydroxylase enzymes that regulate the expression of the hypoxia-inducible factors (HIFs)) [72] and have adapted their functionality through several mechanisms, such as, for example, a heightened degranulation response [73,74]. Since the transcription of effectors is required for HIF-driven processes to adapt to hypoxic environments, HIF-independent pathways might enable neutrophils to adapt to hypoxia more quickly [75,76]. It was established that hypoxia-induced neutrophil degranulation through granule exocytosis increased [76] irrespectively of HIF signalosomes but was reliant on new and little-known protein synthesis [77]. This latter pathway was consistent with the COVID-19 phenotype (similar to ARDS) but was inconsistent with our molecular findings in the COVID-19 patients’ tissues. A nonmacrophage but HIF-independent neutrophil granule exocytosis pathway was unraveled herein with a fan of involved proteins. The molecules and mechanisms implicated in this pathway are unknown, and this study likely shed light on the nodes involved: many proteins identified herein were differentially expressed either exclusively in certain tissues (listed in Table S2) or in all the tissues explored herein (described in Table 1).

In an animal study, preconditioning protected infected and hypoxic mice from morbidity and fatality, observed with acute hypoxia, through the suppression of HIF-driven neutrophil activation (although the degranulation capacity was not assessed) and glucose administration [78]. The hypoxia-enhanced neutrophil degranulation may be lethal, as it may contribute to organ failure, organ damage, and unexpected systemic responses in addition to host infection and local tissue damage [79]. In the literature, a plethora of neutrophils has been established in the lungs [23], the blood [80,81,82,83], and the nasopharyngeal epithelium [84] of severe COVID-19 patients with unspecified characteristics. A transcriptomics-based patient stratification was introduced by Aschenbrenner et al. [85]; the key severity-specific molecules dissected therein were not confirmed by our data analysis. However, this phenomenon is additional proof of the disease’s heterogeneity.

Middleton et al. (2021) [86] explored the neutrophil extracellular traps (NETs) originating from decondensed chromatin released to immobilize SARS-CoV-2 and also able to trigger immunothrombosis. That study suggested that these traps may represent COVID-19 therapeutic targets.

The limitations of our study were mainly due to the following factors:

- The patients’ characteristics (ethnicity, locality, and demographics) and clinical histories (comorbidities and medications administered prior to sampling) were unclear in the source publications.
- The populations were small, and, hence, even if we knew the above characteristics, their stratification into smaller clusters of epidemiological interest would be questioned.
- The hypoxia-enhanced neutrophil degranulation mechanisms’ proteins were little-known.
- The variability in sample preparation could also have impacted the sequence findings.

Future studies should focus on the epidemiology of neutrophil abundance, linking the genomic and clinical data to the epidemiological clusters of patients, in comparison to that of the ones manifesting cytokine storms and macrophage involvement. They should further make an effort to decipher the neutrophil-neglected physiology with focused translational studies.

We believe that our study was the first to provide robust human evidence of ethnicity, locality, and clinical history, filling the gap in the relevant literature. Our investigation, by elucidating the molecules involved, might contribute to the better clinical stratification of COVID-19 patients, including the cytokine and virus entry spike expression, age, ethnicity, and locality dependence of human immune responses to the virus invasion together with the variety of the SARS-CoV-2 variants and the vaccine-induced immunity in a large part of the population [87]. Finally, a wide ongoing scientific field is arising for researchers to elucidate different neutrophil subpopulations and tailor them to current COVID-19 pathology uncertainties and patient heterogeneity.

## 5. Conclusions

In sum, neither macrophage involvement characterized by cytokine storms nor any of the known viral entry cell surface proteins have been detected in any tissue studied herein. On the contrary, immune dysregulation accounted for neutrophil abundance in all the tissues derived from these specific COVID-19 patients under study. This population may involve asymptomatic and/or immunosuppressed patients, children, and adolescents as well as dark-skinned people and/or mountain residents. Our study also disputed the simplicity of neutrophil population functionality, providing a fan of novel nodes to be tailored to unsuspected neutrophil connections and functions in future studies. Finally, our epidemiologic/omics study could contribute to “personalized medicine”.

## Figures and Tables

**Table 1:** Differentially expressed genes common across tissues and their functions.

**Blood ∩ Lung**	**Heart ∩ Bowel**	**Bowel ∩ Lung**	**Heart ∩ Lung**	**Blood ∩ Bowel**
H2BC5, H2BC7, H4C8, IGKV2D-28, IGLV2-14, KIF11, PFKFB3, UPP1	EIF3LP2, IL1RL1, MINCR, RHBDF2, S100A9, TPT1P5, ZBTB42	CD300E, GK, HILPDA, HK2, IER3, PTX3, SNORA71C	FGG, H2AC16, H2AC18, H2AC19, H2AC21, H2BC17, H4C14, H4C2, HSPB6, MT1XP1, SCARNA6, SDCBP2-AS1, TLR2	CD38, G0S2, IGHA1, IGLC2, IL18R1, TDRD9, TIMM10
**Blood ∩ Heart**	**Liver ∩ Lung**	**Liver ∩ Bowel**	**Blood ∩ Heart ∩ Lung**	**Liver ∩ Heart**
HMGB2, IGKC	NDUFB1, RNU6-1016P	BCL2A1, MMP1	MKI67	PSMC1, ZNF524
**Gene Symbol**	**Gene Name**		**Function**	
BCL2A1	BCL2 related protein A1		An apoptosis-related gene and a direct target of the transcription factor NF-κB in inflammatory responses.	
CD300E	CD300e molecule		It encodes a member of the CD300 family of cell surface receptors expressed on myeloid cells, and it is implicated in the modulation of immune responses.	
CD38	CD38 molecule		It is broadly expressed in immune system cells, facilitating effective immune responses.	
EIF3LP2	eukaryotic translation initiation factor 3 subunit L pseudogene 2		Pseudogene with no known function.	
FGG	fibrinogen gamma chain		It encodes a blood-borne glycoprotein suggested to contribute to immune responses.	
G0S2	G0/G1 switch 2		It is associated with cell cycle progression and innate immune response.	
GK	glycerol kinase		It encodes a protein implicated in the regulation of the uptake and metabolism of glycerol that has diverse cellular functions.	
H2AC16	H2A clustered histone 16		The encoded protein constitutes a core component of nucleosomes, and it is expressed in B-cells.	
H2AC18	H2A clustered histone 18		The encoded protein constitutes a core component of nucleosomes, and it is associated with the immune system.	
H2AC19	H2A clustered histone 19		The encoded protein constitutes a core component of nucleosomes, and it is detected in all types of immune cells and is expressed in neutrophils.	
H2AC21	H2A clustered histone 21		The encoded protein constitutes a core component of nucleosomes, and it is detected in many types of immune cells.	
H2BC5	H2B clustered histone 5		The encoded protein constitutes a core component of nucleosomes, and it is detected in all types of immune cells.	
H2BC7	H2B clustered histone 7		The encoded protein constitutes a core component of nucleosomes, and it is detected in some types of immune cells.	
H2BC17	H2B clustered histone 17		The encoded protein constitutes a core component of nucleosomes, and it is expressed in eosinophils.	
H4C2	H4 clustered histone 2		The encoded protein constitutes a core component of nucleosomes, and it is detected in many types of immune cells and is expressed in neutrophils.	
H4C8	H4 clustered histone 8		The encoded protein constitutes a core component of nucleosomes, and it is expressed in eosinophils.	
H4C14	H4 clustered histone 14		The encoded protein constitutes a core component of nucleosomes, and it is detected specifically in neutrophils.	
HILPDA	hypoxia inducible lipid droplet associated		It is implicated in various cellular processes, and it is associated with immune cell infiltration.	
HK2	hexokinase 2		The encoded protein catalyzes the phosphorylation of glucose to glucose-6-phosphate, and it is an innate immune receptor.	
HMGB2	High-mobility group box 2		It encodes a chromatin-associated protein, and it is proposed to be implicated in the innate immune system response to immunogenic nucleic acids.	
HSPB6	heat shock protein family B (small) member 6		It encodes a small heat shock protein which serves as a molecular chaperone.	
IER3	immediate early response 3		It is involved in apoptosis and is associated with aberrant immune response.	
IGHA1	immunoglobulin heavy constant alpha 1		It is linked to receptor-binding activities.	
IGKC	immunoglobulin kappa constant		It participates in antigen binding; it is associated with COVID-19 disease severity.	
IGKV2D-28	immunoglobulin kappa variable 2D-28		Effector phase of humoral immunity modulator.	
IGLC2	immunoglobulin lambda constant 2		Effector phase of humoral immunity modulator.	
IGLV2-14	immunoglobulin lambda variable 2-14		It participates in antigen recognition.	
IL18R1	interleukin 18 receptor 1		It encodes a cytokine receptor necessary for IL18 signaling; it is associated with atherosclerosis.	
IL1RL1	interleukin 1 receptor-like 1		The encoded protein serves as the receptor of IL33; it is associated with fibrosis and heart failure.	
KIF11	kinesin family member 11		It encodes a motor protein implicated in several aspects of spindle dynamics, and it is considered a potential immunological pan-cancer biomarker.	
MINCR	MYC-induced long noncoding RNA		Immune-related long noncoding RNA.	
MKI67	marker of proliferation Ki-67		It encodes a protein essential for cell proliferation, and it is correlated with immune cell infiltration.	
MMP1	matrix metallopeptidase 1		It encodes a protein involved in extracellular matrix degradation in pathophysiological processes, and it is related to cytokine signaling pathways.	
MT1XP1	metallothionein 1X pseudogene 1		Pseudogene with no known function.	
NDUFB1	NADH:ubiquinone oxidoreductase subunit B1		It encodes a protein implicated in the assembly of the mitochondrial respiratory chain complex I; it is involved in inflammatory responses, and it is mainly expressed in monocytes.	
PFKFB3	6-phosphofructo-2-kinase/fructose-2,6-biphosphatase 3		It encodes a protein involved in the synthesis and breakdown of fructose-2,6-bisphosphate.	
PSMC1	proteasome 26S subunit, ATPase 1		It encodes one of the core subunits of the 19S proteasome complex, and it is involved in immune responses and is detected in all types of immune cells.	
PTX3	pentraxin 3		It encodes a member of the pentraxin family of proteins, the expression of which is elicited by inflammatory cytokines.	
RHBDF2	rhomboid 5 homolog 2		It facilitates protein transporter activity, and it is correlated with an immunosuppressive tumor microenvironment.	
RNU6-1016P	RNA, U6 small nuclear 1016, pseudogene		Pseudogene with no known function.	
S100A9	S100 calcium-binding protein A9		It encodes a member of the S100 protein family which is implicated in the regulation of diverse cellular processes; it plays a role in innate immunity and myeloid-derived suppressor-cell-mediated immune suppression.	
SCARNA6	small Cajal-body-specific RNA 6		Small nucleolar RNA which is associated with inflammatory bowel disease.	
SDCBP2-AS1	SDCBP2 antisense RNA 1		Long noncoding RNA.	
SNORA71C	small nucleolar RNA, H/ACA box 71C		Small nucleolar RNA with no known function.	
TDRD9	tudor domain containing 9		It has a potential role in RNA binding activity; it is involved in inflammatory responses and is mainly expressed in monocytes.	
TIMM10	translocase of inner mitochondrial membrane 10		It encodes a component of a protein complex in the mitochondrial intermembrane space.	
TLR2	Toll-like receptor 2		It encodes a member of the family of Toll-like receptors known to play a critical role in the innate immune recognition of pathogens.	
TPT1P5	TPT1 pseudogene 5		Pseudogene with no known function.	
UPP1	uridine phosphorylase 1		The encoded uridine phosphorylase is involved in pyrimidine ribonucleoside salvaging and degradation, and it is linked to immune and inflammatory responses.	
ZBTB42	zinc finger and BTB domain containing 42		It encodes a member of the family of C2H2 zinc finger proteins.	
ZNF524	zinc finger protein 524		The encoded protein is predicted to regulate RNA polymerase II transcription through sequence-specific DNA binding; it is detected in all types of immune cells and is expressed in eosinophils.	

**Table 2:** Genes participating in the neutrophil-mediated immunity process and their corresponding differential expression status in COVID-19. Up represents upregulated; down represents downregulated.

Gene Symbol	Gene Name	Expression
ALDOC	aldolase, fructose-bisphosphate C	Down
CD14	CD14 molecule	Up
CEACAM1	CEA cell adhesion molecule 1	Up
CEACAM3	CEA cell adhesion molecule 3	Up
CYBA	cytochrome b-245 alpha chain	Up
DOCK2	dedicator of cytokinesis 2	Up
ENPP4	ectonucleotide pyrophosphatase/phosphodiesterase 4	Down
FABP5	fatty-acid-binding protein 5	Up
FCGR2A	Fc gamma receptor IIa	Up
GGH	gamma-glutamyl hydrolase	Up
HP	haptoglobin	Up
HVCN1	hydrogen voltage-gated channel 1	Up
IQGAP2	IQ motif-containing GTPase activating protein 2	Up
S100A9	S100 calcium-binding protein A9	Up
TLR2	Toll-like receptor 2	Down
TXNDC5	thioredoxin domain containing 5	Up

## Data Availability

All data and analysis methodologies are contained in the manuscript. Any additional data requests can be addressed to the corresponding authors.

## Supplementary Materials

The following supporting information can be downloaded at https://www.mdpi.com/article/10.3390/v15010104/s1, Table S1: Samples from each gene expression dataset per tissue analyzed in the present study; Table S2: The differentially expressed genes (DEGs) of each of the five transcriptome datasets with the corresponding log_2_FC and FDR values; Table S3: Selected signature COVID-19 gene set.
